# Hypoxia drives shared and distinct transcriptomic changes in two invasive glioma stem cell lines

**DOI:** 10.1038/s41598-024-56102-5

**Published:** 2024-03-27

**Authors:** Valerie J. Marallano, Mary E. Ughetta, Rut Tejero, Sidhanta Nanda, Rohana Ramalingam, Lauren Stalbow, Anirudh Sattiraju, Yong Huang, Aarthi Ramakrishnan, Li Shen, Alexandre Wojcinski, Santosh Kesari, Hongyan Zou, Alexander M. Tsankov, Roland H. Friedel

**Affiliations:** 1 Nash Family Department of Neuroscience, Friedman Brain Institute, Icahn School of Medicine at Mount Sinai, New York, NY 10029 USA; 2 Department of Genetics and Genomic Sciences, Icahn School of Medicine at Mount Sinai, New York, NY 10029 USA; 3 Pacific Neuroscience Institute and Saint John’s Cancer Institute at Providence Saint John’s Health Center, Santa Monica, CA 90404 USA

**Keywords:** Glioblastoma, glioma, hypoxia, Cancer, Cell biology

## Abstract

Glioblastoma (GBM) is the most common primary malignant cancer of the central nervous system. Insufficient oxygenation (hypoxia) has been linked to GBM invasion and aggression, leading to poor patient outcomes. Hypoxia induces gene expression for cellular adaptations. However, GBM is characterized by high intertumoral (molecular subtypes) and intratumoral heterogeneity (cell states), and it is not well understood to what extent hypoxia triggers patient-specific gene responses and cellular diversity in GBM. Here, we surveyed eight patient-derived GBM stem cell lines for invasion phenotypes in 3D culture, which identified two GBM lines showing increased invasiveness in response to hypoxia. RNA-seq analysis of the two patient GBM lines revealed a set of shared hypoxia response genes concerning glucose metabolism, angiogenesis, and autophagy, but also a large set of patient-specific hypoxia-induced genes featuring cell migration and anti-inflammation, highlighting intertumoral diversity of hypoxia responses in GBM. We further applied the Shared GBM Hypoxia gene signature to single cell RNA-seq datasets of glioma patients, which showed that hypoxic cells displayed a shift towards mesenchymal-like (MES) and astrocyte-like (AC) states. Interestingly, in response to hypoxia, tumor cells in IDH-mutant gliomas displayed a strong shift to the AC state, whereas tumor cells in IDH-wildtype gliomas mainly shifted to the MES state. This distinct hypoxia response of IDH-mutant gliomas may contribute to its more favorable prognosis. Our transcriptomic studies provide a basis for future approaches to better understand the diversity of hypoxic niches in gliomas.

## Introduction

Glioblastoma (GBM), the most common and malignant high-grade glioma, has been classified by bulk gene expression to exhibit four transcriptional subtypes^1,2^. Single cell transcriptomics has further revealed intratumoral heterogeneity, with each GBM harboring tumor cells of four distinct differentiation states^3^. Furthermore, mutant forms of the metabolic enzymes IDH1/2 have been identified as molecular drivers of a subset of gliomas previously known as IDH-mutant GBM, now classified as “Astrocytoma, IDH-mutant”^4^. Despite these advances in molecular characterization, GBM continues to carry poor prognosis, with no effective treatment available.

A key environmental factor that drives malignant potency of gliomas is hypoxia (insufficient oxygenation), which occurs in specific niches and has been associated with neovascularization, cell migration, metabolic reprogramming, and poor patient survival^5–7^. Hypoxia leads to stabilization of hypoxia inducible factor 1α (HIF1α), a master transcription factor that controls gene programs for metabolic adaptations, as well as cell specific responses^8^. Part of the hypoxia response is also carried out via a related transcription factor HIF2α, which induces overlapping, but also unique sets of target genes^9^. HIF-independent transcriptional responses to hypoxia may also be driven by epigenetic changes^10^.

The distinct hypoxia activated gene programs for the diverse glioma subtypes are not fully resolved, and a better understanding may help reveal new treatment targets for this devastating disease. In this study, we therefore sought to better characterize the diversity of hypoxia responsive gene programs in gliomas. We first utilized a 3D in vitro invasion assay to identify patient-derived GBM stem cell lines of different subtypes that displayed an increased invasive phenotype in response to hypoxia. We then performed RNA sequencing (RNA-seq) to elucidate shared and patient-specific GBM Hypoxia gene signatures, which featured cell migration and immune-suppression.

We next applied the shared GBM Hypoxia gene signature to recent single-cell RNA-seq (scRNA-seq) data of high-grade glioma patients that included 5 IDH-wildtype and 6 IDH-mutant gliomas^11^. Interestingly, analysis revealed divergent hypoxia responses in IDH-wildtype and IDH-mutant gliomas, with hypoxic glioma cells shifting mainly to a mesenchymal differentiation state in IDH-wildtype, but to an astrocyte-like state in IDH-mutant glioma.

In sum, we reveal patient-specific hypoxia responses in two GBM stem cell lines, and that the mutation status of IDH affects the hypoxia responses in glioma patients. Our findings open the door for further investigations of hypoxia-induced adaptations of glioma cells and of novel targets in the hypoxic subpopulations of gliomas.

## Results

### Distinct migratory responses of patient GBM lines to hypoxia in 3D invasion assay

**Figure 1:**
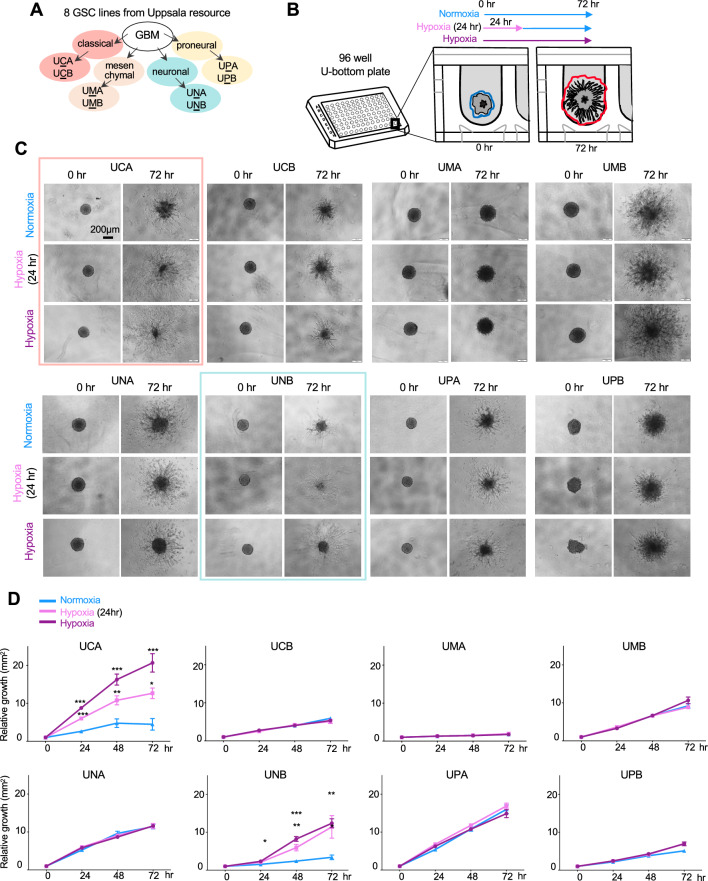
3D matrix invasion assay reveals divergent responses of GBM cells to hypoxia. (**A**) Eight patient-derived GBM stem cell (GSC) lines (Uppsala resource; hgcc.se) with defined subtypes. (**B**) Experimental overview. GSCs were incubated in low-attachment U-well dishes for 3 days to form aggregates. Matrigel was then added to a concentration of 50% to form a 3D matrix around aggregates. Images were taken at 4 h after matrix formation (t = 0 h) and subsequently every 24 h. Culture conditions: normoxia for 72 h; hypoxia for 24 h, followed by normoxia; hypoxia for 72 h. Invasion rates were measured as area inside the outline of the furthest extent of invasion streams (red line in schematic), subtracted by area at t = 0 h (blue line). (**C**) Representative images of GSC aggregates at t = 0 h and t = 72 h in different normoxia/hypoxia conditions. (**D**) Quantification of matrix invasion rates. For each cell line and condition, n = 6–10 aggregates. One-way ANOVA with Tukey correction for comparison against normoxia for each time point; *P < 0.05, **P < 0.01, ***P < 0.001.

We first set out to identify patient GBM lines that would respond to hypoxia by changing their invasive migratory behavior. We utilized a 3D invasion assay to screen eight different patient-derived GBM stem cell lines representing four different transcriptional subtypes from the Human Glioblastoma Cell Culture Resource (Uppsala, Sweden; hgcc.se)^12^ (Fig. 1A). Importantly, these GBM lines had been established in neural stem cell media, which maintains the pathophysiological characteristics of glioma cells, including invasiveness^13–15^.

The 3D invasion assay was conducted by generating GBM cell aggregates in individual U-wells with 50% Matrigel matrix, cultured either under hypoxic (1% O_2_) or normoxic conditions for up to 72 h (Fig. 1B). We also applied transient hypoxia by limiting the hypoxia exposure to the first 24 h, followed by 48 h in normoxia. Invasion rates were determined as the ratio of invasion area relative to the area of aggregates at the beginning of the experiment (Fig. 1B).

Out of the eight GBM lines tested, two lines (UCA and UNB) displayed increased invasion under hypoxia when compared to normoxic conditions (Fig. 1D). Interestingly, the patients from whom these two cell lines were derived had the shortest survival (Table S1), suggesting a link of hypoxia-induced invasiveness and GBM malignancy.

### Hypoxia induces shared and distinct gene sets in patient-derived GBM lines

**Figure 2:**
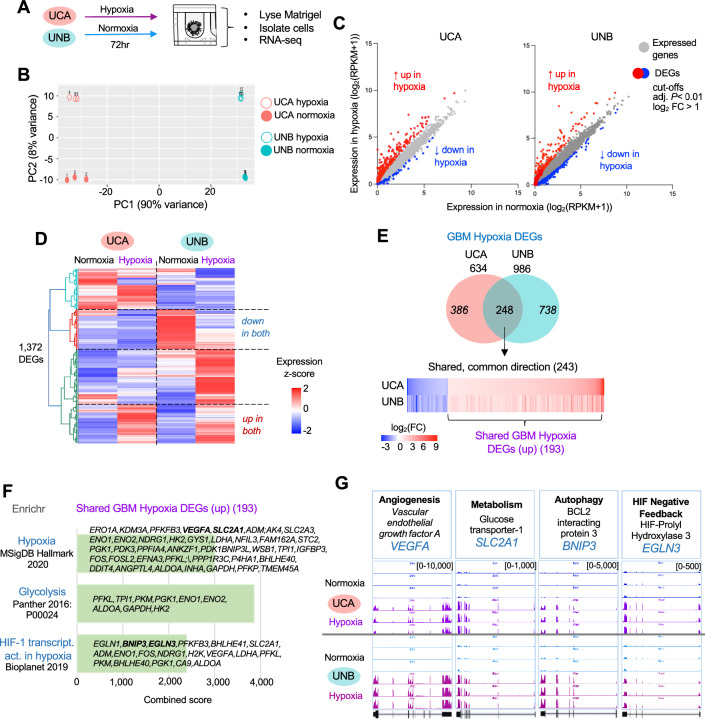
Hypoxia induces a set of shared genes in two GBM lines. (**A**) Experimental approach. Aggregates of GSC lines UCA (classical subtype) and UNB (neural subtype) were cultured for 72 h in 3D matrix under normoxia or hypoxia and then subjected to RNA-seq. (**B**) Principal component (PC) analysis demonstrates clustering of triplicate samples by subtype of neural versus classical (PC1), and hypoxia versus normoxia (PC2). Each RNA-seq sample was pooled from 30 aggregates. (**C**) Expressed genes in UCA and UNB were plotted by log-transformed expression levels in normoxia (x-axis) or hypoxia (y-axis) condition. Hypoxia-induced DEGs are marked in red (upregulated) or blue (downregulated). (**D**) Heatmap of relative gene expression levels showing shared and distinct hypoxia-induced DEGs (cutoff: *P* < 0.01 and log_2_FC > 1) in UCA and UNB. Expression values were row-normalized to calculate z-score. (**E**) Venn diagram showing 248 overlapping hypoxia-induced DEGs in UCA and UNB. After excluding 5 DEGs with opposing directionality, 243 shared DEGs were plotted as heatmap, with 193 genes upregulated. (**F**) Pathway enrichment analysis of Shared GBM Hypoxia DEGs with ENRICHR platform, representative genes are in bold font. (**G**) RNA-seq tracks of representative Shared GBM Hypoxia DEGs in canonical hypoxia response pathways: angiogenesis, metabolism (glycolysis), autophagy, and negative feedback of HIF signaling.

To investigate gene expression changes in the two hypoxia-responsive lines (UCA and UNB), we isolated cells from 3D aggregates cultured under normoxia or hypoxia for 72 h, and RNAs were extracted for Illumina RNA sequencing (Fig. 2A).

Principal component analysis (PCA) of the RNA-seq data revealed close clustering of three independent samples of each condition, confirming reproducibility. The transcriptomic profiles showed that the different GBM lines (UCA vs. UNB) were separated along principal component 1 (PC1), consistent with the intertumoral heterogeneity of GBM. For both lines, the hypoxia exposed cells were consistently distinguishable from their normoxic counterparts along PC2 (Fig. 2B).

Among the differentially expressed genes (DEGs) in response to hypoxia (cut offs: *P* < 0.01 and log_2_FC > 1) (Table S2–S4), a majority was upregulated in both UCA and UNB lines, consistent with HIF functioning as an “on-switch” (Fig. 2C). Heatmap hierarchical clustering analysis of the DEGs illustrated that majority of the genes induced or suppressed in one cell line were not changed in the other line, underscoring intertumoral heterogeneity of GBM in response to hypoxia (Fig. 2D).

An overlap analysis revealed that among the hypoxia DEGs for UCA and UNB (n = 634 and 986, respectively), 248 were shared, among which 193 were upregulated (Fig. 2E). For subsequent studies, we focused on the 193 shared upregulated DEGs, designated as “Shared GBM Hypoxia DEGs”, which were enriched for gene pathways linked to canonical adaptions to hypoxia, e.g., glycolysis and HIF-1 signaling (Fig. 2F). Among them, four major common themes were discernable: angiogenesis, metabolism (glycolysis), autophagy, and HIF negative feedback loop, exemplified by upregulation of the representative genes *VEGFA*, *SLC2A1*, *BNIP3*, and *EGLN3*, respectively (Fig. 2G). We further performed qRT-PCR analysis of these four genes in an independent hypoxia assay in UCA and UNB cells, which confirmed their robust induction under hypoxia (Fig. S1).

### Hypoxia activates patient-specific gene programs in different gliomas

**Figure 3:**
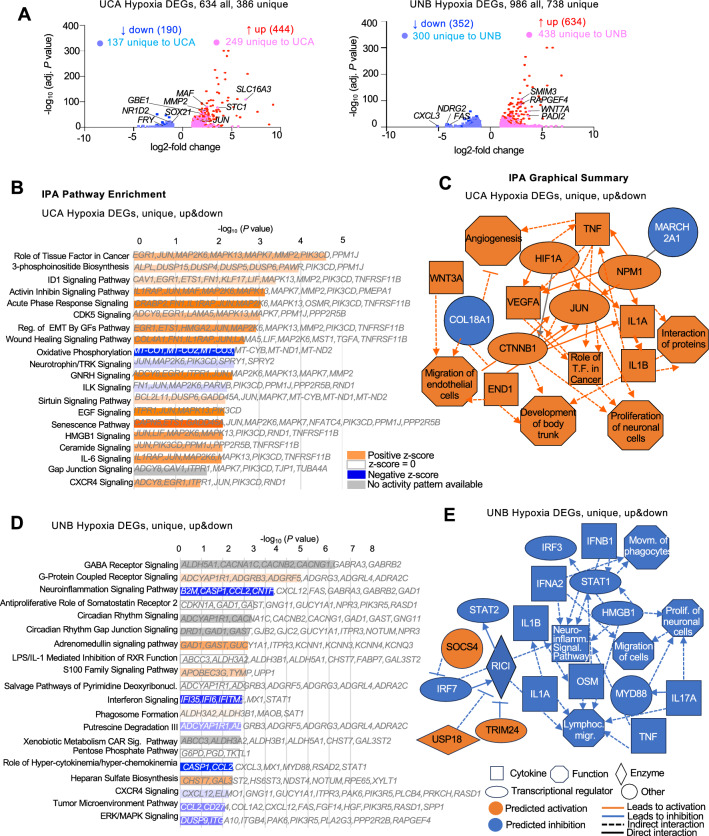
Unique hypoxia response genes of two different GBM cell lines. (**A**) Volcano plots show up- and downregulated DEGs in UCA and UNB under hypoxia. Top unique DEGs are labelled with gene symbol. (**B**,**D**) Ingenuity Pathway Enrichment analysis (IPA) of the DEGs specific for UCA or UNB under hypoxia. (**C**,**E**) IPA graphical summary highlights major biological themes and predicted pathway activation and inhibition for the DEGs specific for UCA or UNB.

To elucidate patient-specific transcriptional responses to hypoxia, we next analyzed hypoxia DEGs unique to UCA or UNB cells. For UCA, 389 of the 634 hypoxia DEGs (61%) were unique, among which 249 were upregulated and 137 downregulated (Fig. 3A). For UNB, 738 of the 986 hypoxia DEGs were unique (75%), and among them, 438 were upregulated and 300 downregulated (Fig. 3A).

Pathway analysis of the patient-specific hypoxia DEGs revealed cell migration and anti-inflammation as main biological themes (described below). For instance, for UCA, specific upregulated hypoxia DEGs included *MMP2* and *STC1*, both linked to invasion and progression of gliomas^16–18^, while specific downregulated DEGs included *NR1D2*, the deficiency of which can promote cancer growth by activating inflammasome^19^, *FRY*, which suppresses the growth promoting transcription factor YAP^20^, and *SOX21*, which acts as a tumor suppressor of gliomas by inhibiting *SOX2* expression and promoting differentiation of glioma cells^21^. On the other hand, for UNB, specific upregulated DEGs included *SMIM3*, a leukemia promoter gene, *WNT7A*, a hypoxia induced EMT driver, *PADI2*, a tumorigenic breast cancer gene, and *RAPGEF4*, which modulates gliomas^22–25^. Downregulated DEGs in UNB included *CXCL3* and *FAS*, which are related to pro-inflammatory action via chemotaxis of neutrophils and apoptotic pathways, respectively^26,27^ (Fig. 3A).

Ingenuity Pathway Analysis (IPA) core enrichment investigation of the UCA-specific hypoxia DEGs also highlighted activation of Tissue Factor pathway, which plays a role in tumor progression and angiogenesis^28^ and has been linked with remodeling of the extracellular matrix and microenvironment of GBM. Enriched pathways also included inhibitor of differentiation 1 (ID1) signaling, which is linked to glioma invasion and suppression of differentiation of GBM stem cells^29^, CDK5 signaling, a well-established regulator of neural progenitor migration^30^ and associated with GBM invasion^31^, and CXCR4 signaling, which emphasizes a migratory response of glioma cells to hypoxia^32^ (Fig. 3B).

We further used the Graphical Summary platform of IPA for patient-specific hypoxia DEGs to construct an overview of predicted relationships among transcription factors, cytokines, and other functional pathways (Fig. 3C). Interestingly, the network of UCA-specific hypoxia DEGs featured the transcription factor JUN as one of its central nodes with links to the HIF1A and VEGFA nodes. JUN is known to drive malignant properties of glioma cells^33^. Other nodes included WNT3A and the canonical WNT-signaling transcription factor β-catenin (CTNNB1). Interestingly, glioma derived WNT3A has been reported to trigger an anti-inflammatory program in microglia, resulting in a less robust and more suppressed immune cell population^34^.

Core IPA analysis of UNB-specific hypoxia DEGs showcased enrichment of the GABA receptor signaling, which has been associated with suppressed proliferation, tumor quiescence, and increased therapy resistance^35^. In addition, there was enrichment for pathways with predicted inhibition of the Neuroinflammation Signaling Pathway and Interferon Signaling, suggesting an immunosuppressive reaction of UNB under hypoxia (Fig. 3D).

The IPA Graphical Summary of UNB-specific hypoxia DEGs highlighted overall downregulation/inhibition of pathways (Fig. 3E), in contrast to the mainly activated pathways of UCA-specific DEGs (see Fig. 3C). This included predicted inhibition of Neuroinflammation Signaling Pathway, movement of phagocytes, and lymphocyte migration. This was paralleled by downregulation/inhibition of nodes for the cytokines and cytokine related proteins IRF7, TNF, MYD88, IL1A and IL1B, which are immune regulators of pro-inflammatory pathways related to interleukin-1 and Toll-like receptor signaling^36–39^.

### IDH mutation status influences the hypoxia response of GBM cells

**Figure 4:**
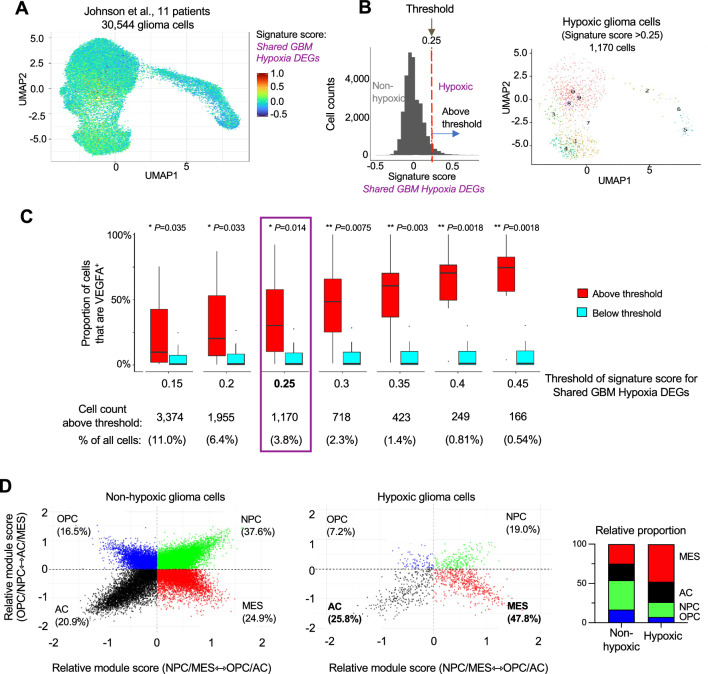
Shared GBM Hypoxia signature suggests hypoxia-induced MES/AC shifts in tumor cells in GBM patients. (**A**) Uniform Manifold Approximation and Projections (UMAP) dimensionality reduction of a scRNA-seq dataset from 11 high-grade glioma patients (Johnson et al.) overlayed with the module score for the gene signature Shared GBM Hypoxia DEGs. (**B**) Left, distribution of signature scores for Shared GBM Hypoxia DEGs for all glioma cells. The selected cut-off threshold for hypoxic glioma cells is indicated by the red dashed line. Right, UMAP of hypoxic glioma cells with signature score > 0.25. (**C**) The proportions of cells expressing VEGFA were plotted as a guide to compare threshold values of gene signature score for Shared GBM Hypoxia DEGs when applied to glioma scRNA-seq dataset from Johnson et al. The threshold value of 0.25, which identified ~ 3.8% of glioma cells as hypoxic, was selected as a cut-off value for subsequent studies. (**D**) Left, two-dimensional representation of the cell states (Neftel et al.) for glioma cells with signature score <  = 0.25 (non-hypoxic) and > 0.25 (hypoxic). Right, stacked bar chart comparing the composition of cell states between non-hypoxic and hypoxic glioma cells show hypoxia-induced shifts towards MES or AC state. AC, astrocyte-like; MES, mesenchymal-like; NPC, neural progenitor cell-like; OPC, oligodendrocyte precursor cell-like state.

To better understand the clinical relevance of the Shared Hypoxia DEGs of UCA and UNB (193 upregulated DEGs), we applied this hypoxia signature across glioma cells in 11 high-grade glioma patients, including 5 IDH-wildtype and 6 IDH-mutant gliomas based on a single cell RNA-seq dataset^11^. For each glioma cell, a signature score for the Shared GBM Hypoxia DEGs was calculated (Fig. 4A). By using the proportion of cells expressing VEGFA as a guide for hypoxia induction, we selected the Shared Hypoxia signature score of 0.25 as a threshold to separate cells into hypoxic versus non-hypoxic, with about 3.8% of GBM cells (1170 out of a total of 30,544) classified as hypoxic (Fig. 4B,C).

To determine the impact of hypoxia on cellular differentiation states (as defined by Neftel and colleagues^3^), we first analyzed the different cell states in each glioma patient (Fig. S2) and then asked if the cell states would be differently represented in hypoxic versus non-hypoxic glioma cells. Interestingly, we found a higher representation of astrocyte-like (AC) and mesenchymal-like (MES) states in hypoxic population, from 20.9 to 25.8% for AC and from 24.9 to 47.8% for MES state, suggesting that hypoxia drives a shift towards AC and MES states. In contrast, the proportion of oligodendrocyte precursor-like (OPC) and neural progenitor-like (NPC) states decreased in hypoxia, from 16.5 to 7.2% for OPC, and from 37.6 to 19.0% for NPC, respectively (Fig. 4D).

**Figure 5:**
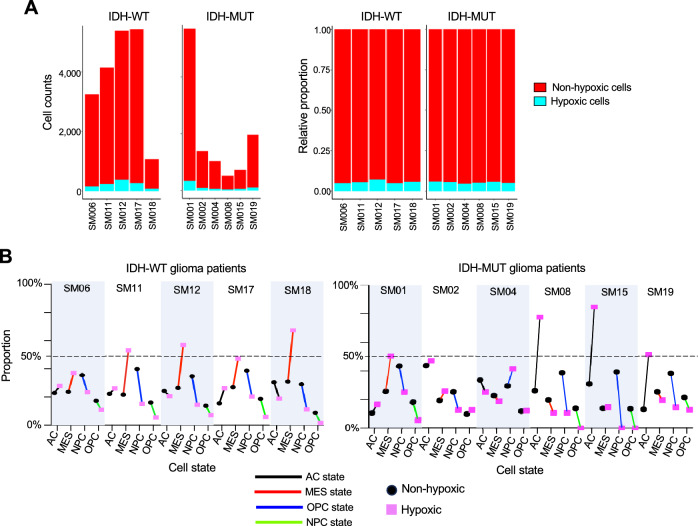
Shifts in glioma cell state are influenced by IDH mutation status. (**A**) The high-grade glioma patients of the study Johnson et al. were stratified into IDH-wildtype and IDH-mutant groups and analyzed for the proportions of hypoxic cells, defined by signature score > 0.25 for the Shared GBM Hypoxia DEGs. Left, absolute cell counts; right, relative proportions. (**B**) Line graphs illustrating shifts of the proportions of cell state between non-hypoxic and hypoxic glioma cells in each of the IDH-WT (5 patients) and IDH-MUT glioma (6 patients).

We next examined the shift of cell states on the level of individual glioma patients according to IDHstatus, as gliomas with IDH1/2 mutation have been defined as a separate entity, with better prognosis among high grade gliomas^40,41^. The relative proportion of the hypoxic cell population was comparable among glioma patients, independent of IDH mutation status, ranging from 1.7 to 5.5% (Fig. 5A). Intriguingly, for IDH-wildtype glioma patients, we observed in all cases a distinct shift of hypoxic cells towards MES state (Fig. 5B), whereas among the IDH-mutant gliomas, the MES shift was largely absent; instead, we detected a strong shift towards an AC state in response to hypoxia (Fig. 5B). As the MES state is associated with higher malignancy and shorter survival^1^, the divergent hypoxia response of IDH-mutant gliomas towards AC state may be part of the molecular underpinnings of their better prognosis.

### Shared GBM Hypoxia DEGs signature is an alternative gene set for analysis of GBM hypoxia

For further confirmation of theShared GBM Hypoxia signature (193 upregulated DEGs), we performed a comparison to the Hallmark Hypoxia signature from the Molecular Signatures Database (MSigDB) (200 genes, compiled from multiple studies^42^). Interestingly, only 20% of the genes from the two sets overlapped (Fig. S3A), indicating again divergent hypoxia gene responses in different cell types. We also applied the MSigDB Hallmark Hypoxia signature to the Johnson et al. patient dataset to separate glioma cells into non-hypoxic and hypoxic cells (Fig. S3B). Analysis of the cell states again confirmed that hypoxia was associated with an enrichment of AC or MES states, with a higher shift of IDH-mutant gliomas to the AC state (Fig. S3C,D).

We also made comparison to a recently defined cancer wide hypoxia signature (HIF metagene) based on genome-wide HIF binding and transcriptome profiling of several cancer cell lines (representing lung, colorectal, liver, prostate, kidney, and breast cancer) under hypoxia^43^. We found that more than half of the HIF metagene signature (26/48) overlapped with our GBM Shared Hypoxia signature (Fig. S4A). We applied the HIF metagene signature to the glioma patient dataset, using a threshold that yielded ~ 3.6% of hypoxic GBM cells, a similar proportion as our Shared GBM Hypoxia signature (Fig. S4B). Analysis of the cell states showed consistent results as above: an overrepresentation of the MES state among hypoxic glioma cells (Fig. S4C). However, an increase in AC state using the HIF metagene was not observed, illustrating our hypoxia signatures unique to glioma (Fig. S4C). When analyzing glioma patients stratified by IDH status, analysis with the HIF metagene consistently identified a significant increase towards the MES state among hypoxic cells of IDH-wildtype patients, and a trend of AC shift among IDH-mutant patients (Fig. S4D). Together, these comparative analyses confirmed that the Shared GBM Hypoxia DEGs signature identified in our study is a valuable alternative for the identification and analysis of hypoxic cells in gliomas.

## Discussion

Glioblastoma is an aggressive brain cancer with high inter-and intratumoral heterogeneity. In this study, we investigated the role of hypoxia as an environmental factor promoting invasiveness and cellular diversity of gliomas. We showed that hypoxia induced both shared and patient-specific gene programs in patient-derived GBM stem cells. While the shared gene set mainly concerned canonical adaptations to hypoxia such as angiogenesis and glycolysis, patient-specific hypoxia genes featured cell migration or immunosuppressive pathways. By applying the Shared GBM Hypoxia signature to a larger single cell RNA-seq patient dataset, we revealed that IDH mutation status influences the responses of glioma cells to hypoxia, with IDH-wildtype glioma mainly shifting to a mesenchymal-like cell state, whereas IDH-mutant gliomas shifting more towards to an astrocyte-like state.

The 3D invasion assay with patient-derived GBM stem cell lines demonstrated that only two out of eight lines, UCA and UNB, displayed a hypoxia-induced increased migratory response. Interestingly, the two patients from whom UCA and UNB were derived had the shortest survival times among the eight patients. Thus, increased migration may not be a general hypoxia response in all glioma patients, but restricted to certain subtypes with more aggressive features. Our bulk RNA-seq analysis showed a clear induction of migration-related genes for the UCA GBM line in response to hypoxia, while immune suppressive pathways were prominent for UNB. Further investigations are needed to reveal the functional significance of these distinct hypoxia-induced gene programs for invasion and the GBM immune landscape.

Our study applied severe hypoxia (1%) to the GBM cells, which triggers a response in which the master transcription factor HIF-1α plays a major role. It should be noted that mild chronic hypoxia (1–5% O_2_) can lead to a switch of the hypoxia responses to one carried out by HIF-2α with potentially divergent functions^44^. Future studies with the 3D aggregate system under mild hypoxia conditions will be informative to investigate a potential HIF1/2 switch in invasive GBM cells.

The shift of hypoxic cells in IDH-wildtype gliomas to a more MES-like state is in alignment with the study by Neftel et al. that characterized the MES-like states of glioma cells as hypoxia dependent^3^. Interestingly, the preferential shift of hypoxic cells to an AC-like state in IDH-mutant gliomas may partly account for the more favorable prognosis of this glioma subtype, and investigations are warranted to elucidate the molecular underpinnings of this difference.

One limitation of the current study is that only two patient-derived GBM lines were sequenced, and additional sequencing of patient samples of diverse GBM molecular subtypes, in particular, sequencing of the six remaining GBM cells lines that did not show increased invasiveness in response to hypoxia would help to compare hypoxia-invasive versus non-invasive glioma transcriptome signatures. Additional information could also be gained by applying 3D tomographic imaging to the GBM aggregates^45^ and application of proliferations markers^46^, which could help to determine if increased GBM invasiveness would go along with reduced proliferation, as has been proposed in the “go or growth” theory^47^. In this context, it is noteworthy that glioblastoma cells do not necessarily follow such dichotomy, as recent studies have demonstrated that invasive GBM cells are often highly proliferative^48^. A further limitation of our study is that invasion assays were conducted in Matrigel, a laminin-rich matrix that does not fully represent the brain extracellular matrix, which is rich in glycosaminoglycans like hyaluronic acid (HA)^49^. Future studies in HA-based 3D matrices (see for example ref.^50^) will present a closer match with the actual matrix conditions of the brain.

Furthermore, the interactions of hypoxic glioma cells with the immune cell environment will need further exploration. This is particularly important in light of the fact that tumor-associated microglia and macrophages (TAMs) can comprise > 30% of the tumor bulk in GBM and contribute to tumor progression and treatment resistance^51^. The distribution of TAMs varies between molecular subtypes of GBM, with the highest TAM content found in GBM of mesenchymal subtype (associated with worst prognosis), and medium and low content in classical and proneural subtypes, respectively^52^. Thus, immune interactions, as suggested by the transcriptomic response of UNB GBM cells to hypoxia, may be a key factor in how hypoxia relates to GBM malignancy. In this context it is noteworthy that our teams have recently characterized the in vivo hypoxia response in a murine GBM model, which featured attraction and sequestration of immune cells inside of hypoxic zones with immunosuppressive gene signatures^53^, a mechanism potentially applicable in human GBM patients.

In sum, our study provides new insights into the molecular strategies that glioma cells exploit to adapt to hypoxia, a key factor of the tumor microenvironment and a driving force of cellular diversity in glioma. Targeting the hypoxia-driven pathways may improve therapies for GBM patients.

## Methods

### GBM stem cell lines

The human GBM stem cell lines UPA (U3047), UPB (U3082), UNA (U3021), UNB (U3085), UMA (U3035), UMB (U3065), UCA (U3056), and UCB (U3086) (all IDH-wildtype) were obtained from the Human Glioblastoma Cell Culture Resource (hgcc.se) of Uppsala University, Sweden, where the cell lines had been established from resected grade 4 GBMs^12^. See Table S1 for additional HGCC cell line information on subtype, gender, age, and survival time. The cell lines were de-identified and no protected patient data was transmitted by HGCC to investigator team. Of note, transcriptional classification of GBM has been updated in 2020 to 3 molecular subtypes^1^, but at the time that the lines were characterized at HGCC four transcriptional subtypes were recognized^2^.

The GBM lines were maintained in neural stem cell media as adherent cultures on laminin-coated dishes, which preserves main physiological characteristics GBM tumor biology^13–15^. Tissue culture dishes were coated with laminin (Invitrogen, 10 µg/ml in PBS) for 1 h at 37 °C. Plates were washed 3 times with PBS before adding human neural stem cell (HNS) media, containing Neurocult NS-A basal medium, Neurocult human NS-A proliferation supplement (Stemcell Technologies), 0.0002% heparin, 10 ng/ml bFGF (Peprotech), and 20 ng/ml EGF (Peprotech). Cells were passaged by dissociation with Accutase (Gibco), incubated for 3 min in 37 °C incubator before dissociation with a micropipette. Two volumes of basal NS-A media were added to dilute Accutase, and cells were pelleted by centrifugation at 1200 rpm in a tabletop centrifuge (Eppendorf Centrifuge 5702) for 3 min, and then resuspended in HNS media before plating.

### 3D invasion assay

We followed the protocol for 3D invasion assay of tumor aggregates as described^54,55^. Briefly, GBM cells were seeded in 96 well low attachment U-shaped bottom plates (Corning) at 2000 cells/well in 100 µl of HNS media. Cells were incubated for 3 days in a cell culture incubator at 37 °C to let them form spheres at the bottom of wells. Next, 50 µl of HNS media was replaced in each well with ice-cold Matrigel (Corning) or the equivalent product Cultrex basement membrane extract (R&D Systems), and after further incubation for 4 h, the gel had solidified (defined as t = 0 h). Photos were taken with a cell culture microscope (Olympus CKX53) at t = 0 h, as well as at t = 24, 48, and 72 h. The perimeter of aggregates at each time point was measured as a ratio to the perimeter of the aggregate in the same well at t = 0 h.

### RNA bulk sequencing

To isolate RNA from cultured GBM aggregates, the solid Matrigel was dissolved by addition of Cultrex Organoid Harvesting Solution (OHS; R&D systems). For each condition, 30 GBM aggregates were collected into a 6-well dish with a 1 ml micropipette with cut off tip from the 96-well U-wells. A volume of 5 ml Cultrex OHS was added and plates were placed for 30 min on a shaker in a 4 °C cold room. Aggregates were collected in a 15 ml tube and pelleted by centrifugation at 1000 rpm for 5 min, then 350 µl RLT buffer of the RNAeasy kit (Qiagen) was added to lyse aggregates by grinding them up with a Dounce homogenizer. The lysed RNA in RLT buffer was stored at − 80 °C until processing.

To prepare cDNA libraries for Illumina sequencing from lysed RNA of GBM aggregates, total RNA was isolated with the RNeasy Micro kit. The cDNA libraries were synthesized with the NEBNext Ultra II Directional RNA Library Prep Kit for Illumina by using 100 ng RNA as input for each replicate sample. Libraries were sequenced at John Wayne Cancer Institute, Santa Monica, on an Illumina HiSeq2500 device with 75 bases single-end reads for a coverage of about 30 million reads per sample.

### Bulk RNA-seq bioinformatic analysis

Raw sequencing reads from UCA and UNB cell lines were mapped to hg38 human genome using HISAT2^56^. Counts of reads mapping to genes were obtained using featureCounts software of Subread package against Ensembl v90 annotation^57^. Differential expression analysis was done using the DESeq2 R package^58^. Differentially expressed genes (DEGs) were defined as genes with log2 fold-change (FC) > 1 between conditions and adjusted *P* value < 0.01. Enrichment analysis of gene sets for biological pathways was performed with the Enrichr platform (https://maayanlab.cloud/Enrichr/)^59^ and the Ingenuity Pathway Analysis (IPA) tool (Qiagen)^60^. The combined score chosen to rank pathways is the multiplication of the odds ratio by the negative natural log of the *P* value of enrichment.

### qRT-PCR of hypoxia induced genes in GBM stem cell lines

UCA and UNB GBM stem cell lines, maintained on laminin-coated dishes, were exposed for 16 h to hypoxic (1% O_2,_ hypoxic chamber) or normoxic control (standard cell culture incubator) conditions. Total RNA was isolated from cells using the RNeasy Plus Mini Kit (Qiagen) and quantified using a Nanodrop device (Thermo Scientific). cDNA was prepared using a Superscript III cDNA synthesis kit (Invitrogen) according to manufacturer’s protocol. Finally, the cDNAs were analyzed for expression levels of hypoxia-induced genes by qRT-PCR on an ABI Prism 7900HT Sequence Detection instrument using PerfeCTa SYBR Green FastMix (Quantabio). Expression of the house keeping gene *HPRT1* was used for normalization of expression levels in each sample.

qRT-PCR primers:

HPRT1-F2: AGATGGTCAAGGTCGCAAG

HPRT1-R2: GTATTCATTATAGTCAAGGGCATATCC

VEGFA-F1: AGTCCAACATCACCATGCAG

VEGFA-R1: TTCCCTTTCCTCGAACTGATTT

SLC2A1-F1: AAAGTGACAAGACACCCGAG

SLC2A1-R1: TGTCAGGTTTGGAAGTCTCATC

BNIP3-F1: GTTCCAGCCTCGGTTTCTATT

BNIP3-R1: AGCCCTGTTGGTATCTTGTG

EGLN3-F1: ATTCATAGCAGATGTGGAGCC

EGLN3-R1: TCAGCATCAAAGTACCAGACAG

### Bioinformatic analysis of glioma patient data

The glioma patient single cell data of the Johnson et al. study^11^ was analyzed using the Seurat package (v4.3.0) as previously described^61^. Briefly, we loaded the Johnson et al. study^11^ count matrix using the read10x function, subset on the glioma cells, and removed low quality cells with fewer than 1000 unique molecular identifiers (UMIs), fewer than 400 detected genes or greater than 25% mitochondrial genes from downstream analysis. Remaining cells were ‘LogNormalized’ using NormalizeData function, and 2000 variable genes were identified using the default ‘vst’ method in the ‘FindVariableFeatures’ function. The normalized data was scaled using the ScaleData function, which was then used to perform principal component (PCA) analysis on the variable gene expression space. Following data integration, Uniform Manifold Approximation and Projection (UMAP) on the first 20 PCAs was performed for visualization of the glioma scRNA-seq data. We then used the AddModuleScore function with default parameters to calculate the enrichment of different hypoxia gene signatures in individual glioma cells.

VEGFA^+^ cells were defined as cells with 2 or more VEGFA unique molecular identifier (UMI) counts. We applied a threshold score of 0.25 for the signature Shared GBM Hypoxia DEGs module (defined in this study; see Table S2 and S5) to separate cells into hypoxic (> 0.25) and non-hypoxic (≤ 0.25) classes. The Lombardi et al. study pan cancer hypoxia gene signature was similarly analyzed on the Johnson et al. scRNA-seq data for comparative analysis. We applied a threshold score of 0.55 for the cancer wide hypoxia signature (HIF metagene) as defined by Lombardi et al. (Table S5) to separate cells into hypoxic (> 0.55) and non-hypoxic (≤ 0.55) classes in similar proportions as classified using the Shared GBM Hypoxia DEGs signature.

Comparative analysis for cell states were visualized by quadrant graphs calculated as described in Neftel et al.^3^. Briefly, scores for each state were calculated using the Seurat AddModuleScore function on gene signatures for MES1/2, AC, OPC, and NPC1/2 states, as defined by Neftel et al.^3^ (Table S5). The cell state with the highest score determined the categorization of the cell (cells in MES1 or MES2 state were summarized as MES, and cells in NPC1 or NPC2 state as NPC).

### Statistical analysis

Statistical analysis of GBM aggregate invasion was performed with GraphPad Prism 9. One-way ANOVA with Tukey correction was applied for comparison of aggregate growth for each time point. Wilcoxon rank-sum test was used for analysis of cell state proportions between patient groups. The GraphPad Prism setting NEJM (New England Journal of Medicine) for reporting of *P* values was applied. *P* ≤ 0.05 was considered as statistically significant (*); *P* ≤ 0.01, **; *P* ≤ 0.001, ***.

### Supplementary Information

Supplementary Figures.

Supplementary Tables.

## Data Availability

The RNA-Seq data has been deposited at the NCBI Gene Expression Omnibus (GEO) database under accession number GSE232725.

## References

[1] Wang Q (2017). Tumor evolution of glioma-intrinsic gene expression subtypes associates with immunological changes in the microenvironment. Cancer Cell.

[2] Verhaak RG (2010). Integrated genomic analysis identifies clinically relevant subtypes of glioblastoma characterized by abnormalities in PDGFRA, IDH1, EGFR, and NF1. Cancer Cell.

[3] Neftel C (2019). An integrative model of cellular states, plasticity, and genetics for glioblastoma. Cell.

[4] Louis DN (2021). The 2021 WHO classification of tumors of the central nervous system: A summary. Neuro Oncol..

[5] Colwell N (2017). Hypoxia in the glioblastoma microenvironment: Shaping the phenotype of cancer stem-like cells. Neuro Oncol..

[6] Monteiro AR, Hill R, Pilkington GJ, Madureira PA (2017). The role of hypoxia in glioblastoma invasion. Cells.

[7] Domenech M, Hernandez A, Plaja A, Martinez-Balibrea E, Balana C (2021). Hypoxia: The cornerstone of glioblastoma. Int. J. Mol. Sci..

[8] Semenza GL (2012). Hypoxia-inducible factors in physiology and medicine. Cell.

[9] Keith B, Johnson RS, Simon MC (2011). HIF1α and HIF2α: Sibling rivalry in hypoxic tumour growth and progression. Nat. Rev. Cancer.

[10] Batie M (2019). Hypoxia induces rapid changes to histone methylation and reprograms chromatin. Science.

[11] Johnson KC (2021). Single-cell multimodal glioma analyses identify epigenetic regulators of cellular plasticity and environmental stress response. Nat. Genet..

[12] Xie Y (2015). The human glioblastoma cell culture resource: Validated cell models representing all molecular subtypes. EBioMedicine.

[13] Lee J (2006). Tumor stem cells derived from glioblastomas cultured in bFGF and EGF more closely mirror the phenotype and genotype of primary tumors than do serum-cultured cell lines. Cancer Cell.

[14] Pollard SM (2009). Glioma stem cell lines expanded in adherent culture have tumor-specific phenotypes and are suitable for chemical and genetic screens. Cell Stem Cell.

[15] Galli R (2004). Isolation and characterization of tumorigenic, stem-like neural precursors from human glioblastoma. Cancer Res..

[16] Kargiotis O (2008). Adenovirus-mediated transfer of siRNA against MMP-2 mRNA results in impaired invasion and tumor-induced angiogenesis, induces apoptosis in vitro and inhibits tumor growth in vivo in glioblastoma. Oncogene.

[17] Rahme GJ, Israel MA (2015). Id4 suppresses MMP2-mediated invasion of glioblastoma-derived cells by direct inactivation of Twist1 function. Oncogene.

[18] Xiong Y, Wang Q (2019). STC1 regulates glioblastoma migration and invasion via the TGF-β/SMAD4 signaling pathway. Mol. Med. Rep..

[19] Kim SM, Jeon Y, Jang JY, Lee H (2023). NR1D1 deficiency in the tumor microenvironment promotes lung tumor development by activating the NLRP3 inflammasome. Cell Death Discov..

[20] Irie K, Nagai T, Mizuno K (2020). Furry protein suppresses nuclear localization of yes-associated protein (YAP) by activating NDR kinase and binding to YAP. J. Biol. Chem..

[21] Stevanovic M, Kovacevic-Grujicic N, Mojsin M, Milivojevic M, Drakulic D (2021). SOX transcription factors and glioma stem cells: Choosing between stemness and differentiation. World J. Stem Cells.

[22] Liu Y (2022). Downregulation of SMIM3 inhibits growth of leukemia via PI3K-AKT signaling pathway and correlates with prognosis of adult acute myeloid leukemia with normal karyotype. J. Transl. Med..

[23] Richard SA (2020). EPAC2: A new and promising protein for glioma pathogenesis and therapy. Oncol. Rev..

[24] Wang H (2016). PADI2 gene confers susceptibility to breast cancer and plays tumorigenic role via ACSL4, BINC3 and CA9 signaling. Cancer Cell Int..

[25] Wu DJ (2018). High expression of WNT7A predicts poor prognosis and promote tumor metastasis in pancreatic ductal adenocarcinoma. Sci. Rep..

[26] Gratas C (1997). Fas ligand expression in glioblastoma cell lines and primary astrocytic brain tumors. Brain Pathol..

[27] Blengio F (2013). The hypoxic environment reprograms the cytokine/chemokine expression profile of human mature dendritic cells. Immunobiology.

[28] Kasthuri RS, Taubman MB, Mackman N (2009). Role of tissue factor in cancer. J. Clin. Oncol..

[29] Soroceanu L (2013). Id-1 is a key transcriptional regulator of glioblastoma aggressiveness and a novel therapeutic target. Cancer Res..

[30] Beffert U (2004). Reelin and cyclin-dependent kinase 5-dependent signals cooperate in regulating neuronal migration and synaptic transmission. J. Neurosci..

[31] Liu R (2008). Cdk5-mediated regulation of the PIKE-A-Akt pathway and glioblastoma cell invasion. Proc. Natl. Acad. Sci. USA.

[32] Zagzag D (2008). Hypoxia- and vascular endothelial growth factor-induced stromal cell-derived factor-1alpha/CXCR4 expression in glioblastomas: One plausible explanation of Scherer's structures. Am. J. Pathol..

[33] Blau L (2012). Aberrant expression of c-Jun in glioblastoma by internal ribosome entry site (IRES)-mediated translational activation. Proc. Natl. Acad. Sci. USA.

[34] Matias D (2019). GBM-derived Wnt3a induces M2-like phenotype in microglial cells through Wnt/beta-catenin signaling. Mol. Neurobiol..

[35] Blanchart A (2017). Endogenous GABAA receptor activity suppresses glioma growth. Oncogene.

[36] Gabay C, Lamacchia C, Palmer G (2010). IL-1 pathways in inflammation and human diseases. Nat. Rev. Rheumatol..

[37] Wei Q (2021). TNFalpha secreted by glioma associated macrophages promotes endothelial activation and resistance against anti-angiogenic therapy. Acta Neuropathol. Commun..

[38] Jantsch J (2011). Toll-like receptor activation and hypoxia use distinct signaling pathways to stabilize hypoxia-inducible factor 1alpha (HIF1A) and result in differential HIF1A-dependent gene expression. J. Leukoc. Biol..

[39] Qing F, Liu Z (2023). Interferon regulatory factor 7 in inflammation, cancer and infection. Front. Immunol..

[40] Georgescu MM (2021). Multi-platform classification of IDH-wild-type glioblastoma based on ERK/MAPK pathway: Diagnostic. Progn. Ther. Implic. Cancers (Basel).

[41] Han S (2020). IDH mutation in glioma: Molecular mechanisms and potential therapeutic targets. Br. J. Cancer.

[42] Liberzon A (2015). The molecular signatures database (MSigDB) hallmark gene set collection. Cell Syst..

[43] Lombardi O (2022). Pan-cancer analysis of tissue and single-cell HIF-pathway activation using a conserved gene signature. Cell Rep..

[44] Koh MY, Powis G (2012). Passing the baton: The HIF switch. Trends Biochem Sci..

[45] Ozturk MS (2020). High-resolution tomographic analysis of in vitro 3D glioblastoma tumor model under long-term drug treatment. Sci. Adv..

[46] Tejero R (2019). Gene signatures of quiescent glioblastoma cells reveal mesenchymal shift and interactions with niche microenvironment. EBioMedicine.

[47] Odde DJ (2023). Glioblastoma cell invasion: Go? Grow? Yes. Neuro Oncol..

[48] Ratliff M (2023). Individual glioblastoma cells harbor both proliferative and invasive capabilities during tumor progression. Neuro Oncol..

[49] Rape A, Ananthanarayanan B, Kumar S (2014). Engineering strategies to mimic the glioblastoma microenvironment. Adv. Drug Deliv. Rev..

[50] Chen J (2020). Suppression of LIM kinase 1 and LIM kinase 2 limits glioblastoma invasion. Cancer Res..

[51] Hambardzumyan D, Gutmann DH, Kettenmann H (2016). The role of microglia and macrophages in glioma maintenance and progression. Nat. Neurosci..

[52] Keane L, Cheray M, Blomgren K, Joseph B (2021). Multifaceted microglia—Key players in primary brain tumour heterogeneity. Nat. Rev. Neurol..

[53] Sattiraju A (2023). Hypoxic niches attract and sequester tumor-associated macrophages and cytotoxic T cells and reprogram them for immunosuppression. Immunity.

[54] Nagaraja S (2017). Transcriptional dependencies in diffuse intrinsic pontine glioma. Cancer Cell.

[55] Vinci M, Box C, Eccles SA (2015). Three-dimensional (3D) tumor spheroid invasion assay. J. Vis. Exp.

[56] Kim D, Langmead B, Salzberg SL (2015). HISAT: A fast spliced aligner with low memory requirements. Nat. Methods.

[57] Liao Y, Smyth GK, Shi W (2019). The R package Rsubread is easier, faster, cheaper and better for alignment and quantification of RNA sequencing reads. Nucleic Acids Res..

[58] Love MI, Huber W, Anders S (2014). Moderated estimation of fold change and dispersion for RNA-seq data with DESeq2. Genome Biol..

[59] Xie Z (2021). Gene set knowledge discovery with Enrichr. Curr. Protoc..

[60] Krämer A, Green J, Pollard J, Tugendreich S (2014). Causal analysis approaches in ingenuity pathway analysis. Bioinformatics.

[61] Zhao, W. *et al.* A cellular and spatial atlas of TP53-associated tissue remodeling in lung adenocarcinoma. *bioRxiv.*10.1101/2023.06.28.546977 (2024). 10.1038/s43018-025-01053-7 PMC12883129 41057692

