# Supplementary material for: Hypoxia drives shared and distinct transcriptomic changes in two invasive glioma stem cell lines
Source: Sci Rep. 2024 Mar 27;14:7246. doi: 10.1038/s41598-024-56102-5 (PMC10973515; doi:10.1038/s41598-024-56102-5)
Supplement: Supplementary file 1 — Supplementary Figures. [file 41598_2024_56102_MOESM1_ESM.pdf]

Fig. S1

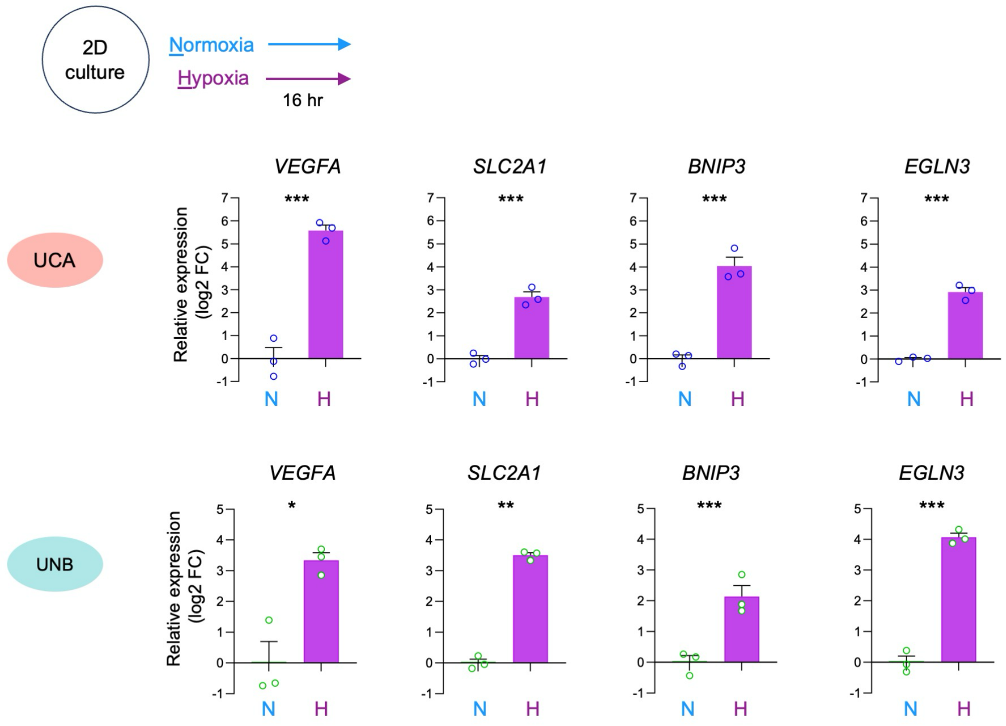

Supplemental Figure S1. qRT-PCR confirms induction of shared hypoxia genes in UCA and UNB GBM cells.

UCA and UNB cells were cultured in laminin-coated dishes for 16 hours under normoxia or hypoxia (1% O_2). qRT-PCR analysis confirmed induction of the shared hypoxia response genes vascular endothelial growth factor A (*VEGFA*), solute carrier family 2 member 1 (*SLC2A1*), Bcl-2 interacting protein 3 (*BNIP3*), and Egl-9 Family Hypoxia Inducible Factor 3 (*EGLN3*). Expression values were normalized using the *HPRT1* gene. Unpaired two-tailed Student's *t*-test. *, $P \leq 0.05$; **, $P \leq 0.01$; ***, $P \leq 0.001$.

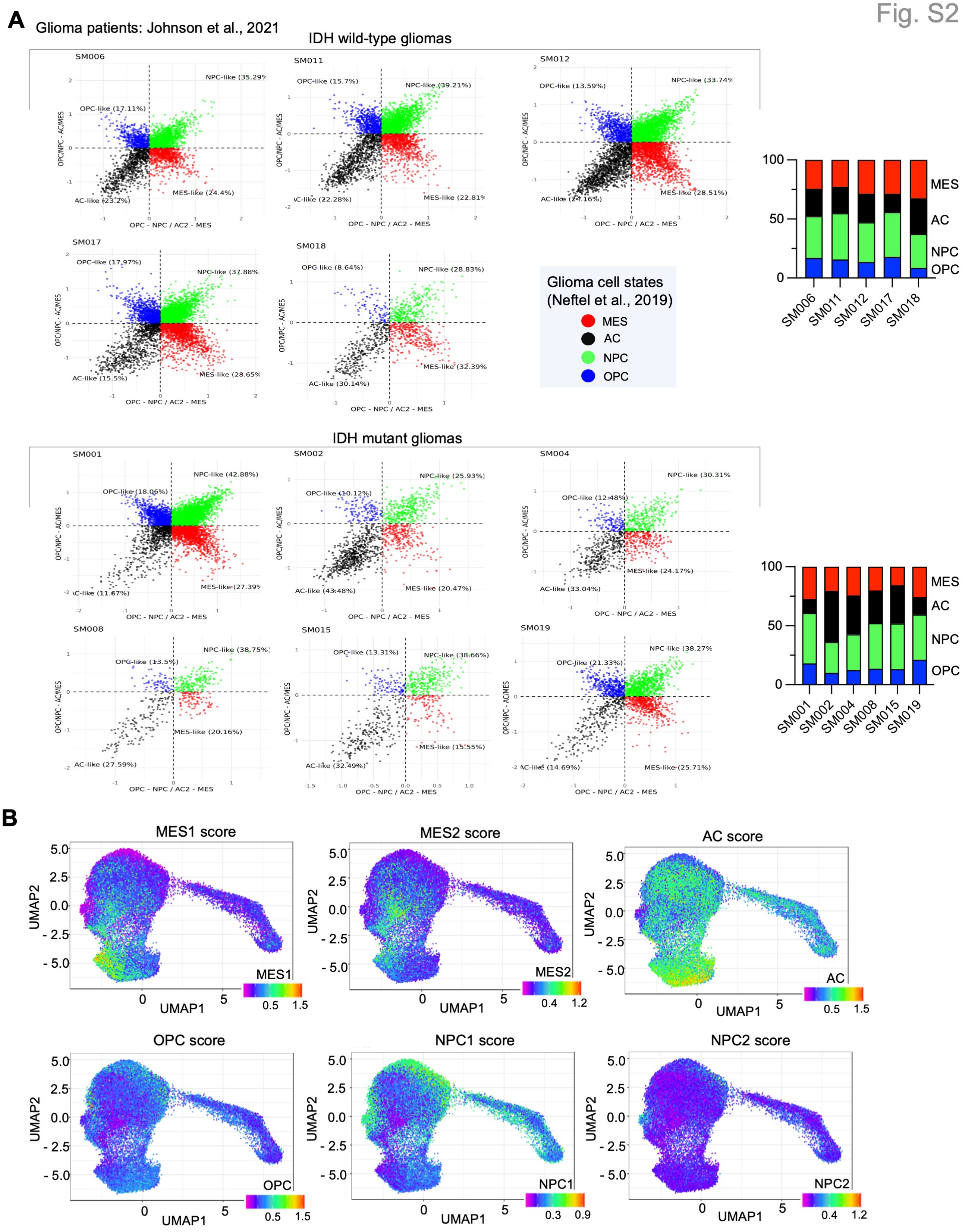

Supplemental Figure S2. Representation of different cancer cell states in high-grade glioma patients.

A) High-grade glioma patients from Johnson et al. were analyzed for relative distribution of cell states

(Neftel et al.) among malignant cells. AC, astrocyte-like; MES, mesenchymal-like; NPC, neural progenitor cell-like; and OPC, oligodendrocyte precursor cell-like state.

B) UMAP representation of all glioma cells from Johnson et al., overlayed with signature scores for different cell states as defined in Neftel et al..

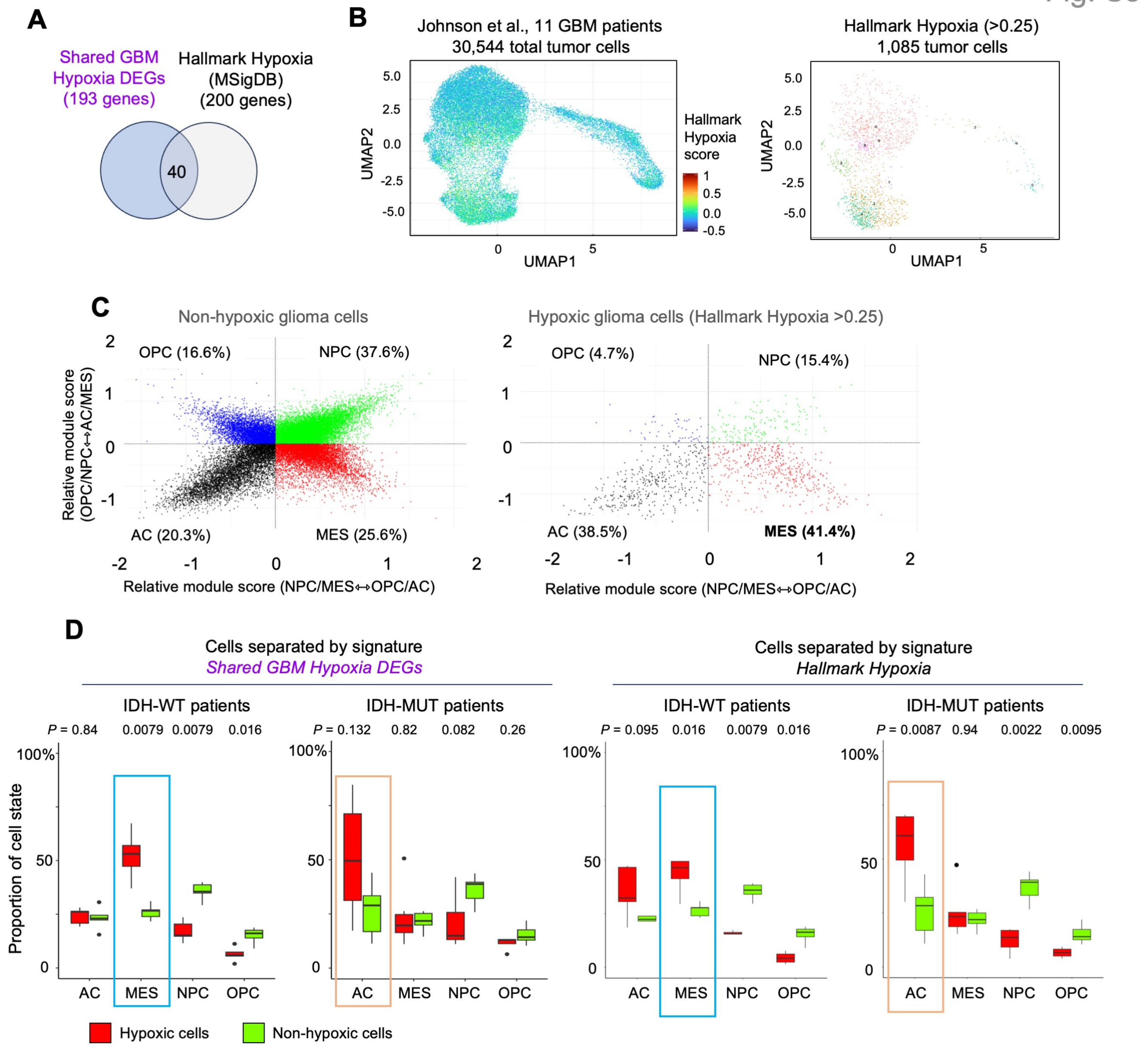

Supplemental Figure S3. Comparison of Shared GBM Hypoxia DEGs with Hallmark Hypoxia signature.

A) Venn diagram illustrating partial overlap of Shared GBM Hypoxia DEGs (this study) with Hallmark Hypoxia gene set (MSigDB database).

B) Left, UMAP dimensionality reduction of glioma cells from scRNA-seq dataset Johnson et al., overlayed with signature score for Hallmark Hypoxia gene set. Right, subset of hypoxic glioma cells (signature score >0.25).

C) Representation of cell states (Neftel et al.) for glioma cells with Hallmark Hypoxia module score <=0.25 (non-hypoxic; left) and >0.25 (hypoxic; right). AC, astrocyte-like; MES, mesenchymal-like; NPC, neural progenitor cell-like; and OPC, oligodendrocyte precursor cell-like state.

D) Box plots illustrating the cell state proportions in non-hypoxic vs. hypoxic glioma cells (Johnson et al. dataset). For graph on the left, the Shared GBM Hypoxia DEGs signature was applied to define hypoxic cells, and for the graph on the right, the Hallmark Hypoxia signature was applied. Boxplots span the range from the first to the third quartile, whiskers extend up to 1.5 times of interquartile range; outliers indicated by dots. n = 5 for IDH-WT patients and n=6 for IDH-mutant patients; Wilcoxon rank-sum test. Blue outline denotes hypoxia-driven MES-like shift in IDH-WT patients, orange outline denotes hypoxia-driven AC-like shift in IDH-MUT patients.

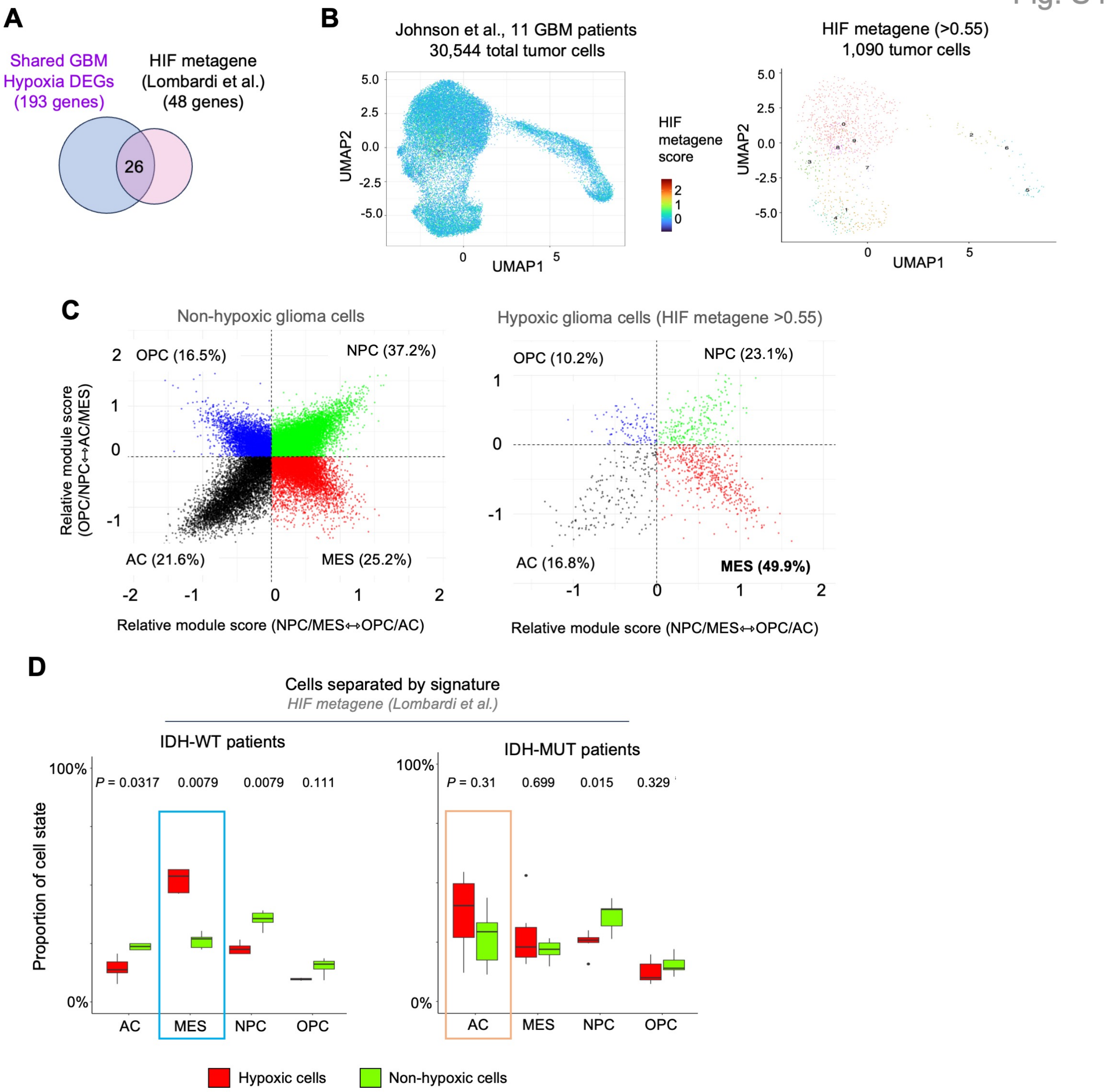

Supplemental Figure S4. Application of HIF metagene signature (Lombardi et al.) to glioma patients.

A) Venn diagram illustrating partial overlap of genes of Shared GBM Hypoxia DEGs with the HIF metagene signature (Lombardi et al.).

B) Left, UMAP dimensionality reduction of glioma cells from scRNA-seq dataset Johnson et al., overlayed with scores for HIF metagene signature. Right, subset of hypoxic glioma cells (signature score >0.55), which yielded a similar proportion of hypoxic cells as the Shared GBM Hypoxia signature (this study).

C) Representation of cell states (Neftel et al.) for glioma cells with HIF metagene score <=0.55 (non-hypoxic; left) and >0.55 (hypoxic; right). AC, astrocyte-like; MES, mesenchymal-like; NPC, neural progenitor cell-like; and OPC, oligodendrocyte precursor cell-like state.

D) Box plots illustrating the cell state proportions in non-hypoxic and hypoxic glioma cells (Johnson et al. dataset) when the HIF metagene signature was applied. Boxplots span the range from the first to the third quartile, whiskers extend up to 1.5 times of interquartile range; outliers indicated by dots. n = 5 for IDH-WT patients and n=6 for IDH-mutant patients; Wilcoxon rank-sum test. Blue outline denotes hypoxia-driven MES-like shift in IDH-WT patients, dashed orange outline denotes a trend of hypoxia-driven AC-like shift in IDH-MUT patients.
